# Highly selective inhibition of Bruton’s tyrosine kinase attenuates skin and brain disease in murine lupus

**DOI:** 10.1186/s13075-017-1500-0

**Published:** 2018-01-25

**Authors:** Samantha A. Chalmers, Jing Wen, Jessica Doerner, Ariel Stock, Carla M. Cuda, Hadijat M. Makinde, Harris Perlman, Todd Bosanac, Deborah Webb, Gerald Nabozny, Jay S. Fine, Elliott Klein, Meera Ramanujam, Chaim Putterman

**Affiliations:** 1 Department of Microbiology and Immunology, Albert Einstein College of Medicine, Bronx, NY USA; 2 Division of Rheumatology, Northwestern University Feinberg School of Medicine, Chicago, IL USA; 3 Small Molecule Discovery Research, Boehringer Ingelheim Pharmaceuticals, Ridgefield, CT USA; 4 Immunology and Respiratory Disease Research, Boehringer Ingelheim Pharmaceuticals, Ridgefield, CT USA; 5 Division of Rheumatology, Albert Einstein College of Medicine, F701N, 1300 Morris Park Ave, Bronx, NY 10461 USA

**Keywords:** Systemic lupus erythematous (SLE), Neuropsychiatric lupus (NPSLE), Cutaneous lupus erythematosus (CLE), Bruton’s tyrosine kinase (BTK)

## Abstract

**Background:**

Systemic lupus erythematosus (SLE) is a systemic autoimmune disease that affects different end organs, including skin and brain. We and others have previously shown the importance of macrophages in the pathogenesis of cutaneous and neuropsychiatric lupus. Additionally, autoantibodies produced by autoreactive B cells are thought to play a role in both the skin and central nervous system pathologies associated with SLE.

**Methods:**

We used a novel inhibitor of Bruton’s tyrosine kinase (BTK), BI-BTK-1, to target both macrophage and B cell function in the MRL-lpr/lpr murine model of SLE, and examined the effect of treatment on skin and brain disease.

**Results:**

We found that treatment with BI-BTK-1 significantly attenuated the lupus associated cutaneous and neuropsychiatric disease phenotypes in MRL/lpr mice. Specifically, BI-BTK-1 treated mice had fewer macroscopic and microscopic skin lesions, reduced cutaneous cellular infiltration, and diminished inflammatory cytokine expression compared to control mice. BTK inhibition also significantly improved cognitive function, and decreased accumulation of T cells, B cells, and macrophages within the central nervous system, specifically the choroid plexus.

**Conclusions:**

Directed therapies may improve the response rate in lupus-driven target organ involvement, and decrease the dangerous side effects associated with global immunosuppression. Overall, our results suggest that inhibition of BTK may be a promising therapeutic option for cutaneous and neuropsychiatric disease associated with SLE.

**Electronic supplementary material:**

The online version of this article (doi:10.1186/s13075-017-1500-0) contains supplementary material, which is available to authorized users.

## Background

Systemic lupus erythematosus (SLE) is a multifactorial autoimmune disease, which results in different end organ pathologies including prominent skin and neuropsychiatric manifestations [1]. Cutaneous disease, known as cutaneous lupus erythematosus (CLE), occurs in up to 75% of patients and has a profound effect on the patient’s quality of life [2]. Neuropsychiatric SLE (NPSLE) occurs in about 40% of patients; brain involvement manifests through a diverse array of signs and symptoms including seizures, psychosis, cognitive dysfunction, and depression, and has significant prognostic implications [3]. Current therapies for both cutaneous and neuropsychiatric manifestations are associated with multiple side effects ranging from mild to severe (e.g. increased risk of infection and cancer associated with immunosuppressive agents). These less than ideal side-effect profiles of current therapies limit their use. As such, patients with SLE would benefit from the development of more targeted treatment options with improved side-effect profiles, and perhaps increased efficacy as well [4]. In SLE, cutaneous and brain manifestations can appear alone, without the involvement of other target organs (e.g. kidney). Moreover, CLE can be an isolated disease entity, outside the context of systemic lupus. Finally, therapeutic approaches to one particular organ manifestation in SLE are not necessarily applicable to disease in other organs. Based on all of these considerations, there remains a pressing need to develop therapies for non-renal lupus manifestations as well.

Two major cell types contributing to SLE pathogenesis are B cells and macrophages [1]. B cells produce autoantibodies that localize to lupus target organs, including both the skin and brain in patients with CLE [5] and NPSLE [6], respectively. In CLE, immune complexes are deposited at the dermo-epidermal junction where they can activate local Fc-receptor-bearing cells, initiating an inflammatory cascade with the potential to cause local damage [2]. Similarly, in NPSLE autoantibodies can be deposited in the brain after disruption of the blood brain barrier, promoting inflammation and cellular damage. Activated macrophages are also found infiltrating into these tissues with active inflammation, and their effector functions are thought to be important to disease pathogenesis [7–10].

Bruton’s tyrosine kinase (BTK) is important for both B cell and macrophage function [11]. Specifically, BTK is vital for B cell development, survival, and function (e.g. B cell receptor (BCR) and toll-like receptor (TLR) signaling), while in macrophages BTK mediates Fc receptor and TLR signaling and macrophage polarization [11–14]. These pivotal roles for BTK in both B cells and macrophages indicate that this enzyme could potentially be a valuable therapeutic target in different end-organ pathologies in SLE.

BI-BTK-1 is a novel, potent, highly selective, and irreversible inhibitor of BTK, which we previously have shown to directly inhibit B cell and myeloid cell activation by in vitro BCR and FcγR stimulation, respectively [15]. Furthermore, we have found that BI-BTK-1 can prevent kidney disease in nephrotoxic serum nephritis [15]. However, this particular inducible model of immune-complex mediated nephritis is short term (1–2 weeks), and only a single organ (the kidney) is affected. A more challenging and clinically relevant assessment of the potential of BTK inhibition to modulate lupus would be in administration of an inhibitor over a prolonged period of time, in a lupus model with multi-organ involvement.

MRL/lpr mice, which are characterized genetically by defective Fas-mediated apoptosis, exhibit spontaneous systemic autoimmune disease that mimics human SLE, including predominance in female animals, circulating nuclear auto-antibodies, and pathology in multiple end organs. In addition to the development of spontaneous skin lesions with clinical and histopathologic characteristics resembling those seen in human SLE, disease progression in the MRL/lpr strain is associated with cognitive dysfunction and depression-like behavior. Therefore, while defects in *Fas* are not causative in human lupus, the considerations described make the MRL/lpr strain an excellent and often-used lupus model, not just for the study of lupus nephritis, but also of CLE and NPSLE [16, 17]. For this study, we assessed the effect of BI-BTK-1 treatment in cutaneous and neuropsychiatric manifestations in the MRL/lpr mouse model. We treated mice with BI-BTK-1 and examined the development of spontaneous skin lesions and behavioral abnormalities, to investigate if BTK represents a potential therapeutic target for these classic but often treatment-resistant lupus target organ manifestations.

## Methods

### Mice

Female MRL/MpJ-Fas^*lpr*^/J (MRL/lpr) mice (3–4 weeks old) were purchased from the Jackson Laboratory (Bar Harbor, ME, USA), and housed and aged at the Albert Einstein College of Medicine animal facility (Bronx, NY, USA). Once the mice were 8–9 weeks of age, mice were started on medicated chow that provided a daily dose of ~ 10 mg/kg of BI-BTK-1 [15] (n = 12), or comparable control chow (n = 12). BI-BTK-1 was synthesized and provided by Boehringer Ingelheim. The mice were kept on the chow until the time of sacrifice. Monitoring for skin lesions was started at 12–13 weeks of age, and the mice underwent behavioral testing at 17–18 weeks of age. All available mice, or for technical considerations a randomly selected subset, were evaluated in the studies detailed subsequently. Animal studies were approved by the institutional animal care committee.

### Histopathologic assessment

At the time of killing, mice were perfused with ice cold PBS. Lesional skin and brains were harvested, fixed, paraffin-embedded, and sectioned and stained at the Histology and Comparative Pathology Core at the Albert Einstein College of Medicine. Sections were also stained with hematoxylin and eosin (H&E).

Skin sections were blindly scored based upon a system we have described previously [9]. Briefly, skin sections were assigned two separate scores. The first was for the epidermis (0–5, in increments of 0.5) based upon the severity of interface dermatitis and the thickening of the epidermis. The second score was assigned to the dermis (0–3, in increments of 0.5), scoring the amount of infiltrating cells. The scores were added together providing a total skin score for each mouse, ranging from 0 to 8. Brain sections were assessed for choroid plexus infiltration at the level of the dorsal fourth ventricle, and blindly scored based upon stromal expansion and cellular infiltration (ranging from 0 to 4).

### Assessment of macroscopic skin lesions

Macroscopic skin lesions were scored blindly by trained observers every 2–3 weeks starting at 12–13 weeks of age, as previously described [9]. Multiple body regions were assessed, and assigned a numerical value based upon erythema, alopecia, skin thickening, and scaling. The scores were adjusted for the degree of involvement and the percent of body surface covered. A score was then assigned to each mouse, ranging from 0 to 72 [9].

### Immunofluorescent staining

Sections were deparaffinized and rehydrated, and subjected to antigen retrieval in a citrate buffer (pH 6) for 5 minutes at 90 °C. The sections were then cooled to room temperature, washed three times with PBS, and incubated for 1 hour at room temperature in blocking buffer (20% normal horse serum, 0.5% Triton, in PBS). Primary antibodies were then added and were incubated at 4 °C overnight. After 14–16 hours of incubation, the slides were washed three times with PBS and incubated with the appropriate fluorescently labelled secondary antibodies for 1 hour at room temperature. The slides were then washed, stained with 4′,6-diamid ino-2-phenylindole (DAPI), mounted, and imaged. Specifically, we simultaneously stained sections for multiple different markers. The first stain panel combined rabbit anti-IBA-1 (1:250, Wako); rat anti-CD4 (1:100, Ebioscience); and AF647 conjugated donkey anti-mouse IgG (1:500, Jackson ImmunoResearch Labs, West Grove, PA, USA). For this stain, the secondary antibodies used were donkey anti-rabbit AF488 (1:200) and donkey anti-rat AF594 (1:200) (Jackson ImmunoResearch). The second stain panel used both rabbit anti-IBA-1 and rat anti-Mac2 (both at 1:100, Ebioscience), with anti-rabbit AF488 and anti-rat AF594 as secondary antibodies (Jackson ImmunoResearch). A third stain panel contained rat anti-CD4 (1:100, Ebioscience); rabbit anti-B220 (1:100, Affymetrix); and goat anti-C3 (1:100, MP Biomedicals). For this stain, the secondary antibodies used were donkey anti-rat AF 594, donkey anti-rabbit AF488, and donkey anti-goat AF647 (all 1:100, Jackson ImmunoResearch). Finally, the fourth stain panel contained rat anti-fibronectin (1:100, Abcam); goat anti-albumin (1:100, Bethyl Laboratories); and AF647 conjugated donkey anti-mouse IgG (1:500, Jackson Immuno Research). For this stain, the secondary antibodies were donkey anti-rat AF488 and donkey anti-goat AF594 (1:100, Jackson ImmunoResearch). To stain for neutrophils, we stained skin and brain sections with rat anti-Ly6G (1:100, BD-Bioscience), and used donkey anti-rat AF488 (1:100, Jackson ImmunoResearch) as the secondary antibody. For assessment of apoptotic cells in paraffin tissue, the In Situ Cell Death Detection Kit, Fluorescein (Sigma) was used according to the manufacturer’s instructions.

### Behavioral testing

MRL/lpr mice spontaneously display a neuropsychiatric phenotype, including cognitive abnormalities (memory deficits) and depression-like behavior. To assess the effects of BI-BTK-1 on brain disease, neurobehavioral testing was performed as previously described [18]. Briefly, in the object placement test mice were exposed to two identical objects in different locations within an arena for 5 minutes. The mice were then removed, and after a 25-minute retention interval returned to the testing arena, where one of the objects had been moved to a novel location. The relative time the mouse spent exploring the objects was measured and the percent preference calculated. In the object recognition test, the mice were put into an arena and allowed to explore two identical objects for 3 minutes. Mice were then removed, subjected to a 90-minute retention period and returned to the arena, where one of the objects had been replaced with a novel object. The relative time spent with the objects was measured, and the percent preference was calculated. Cognitively normal mice (i.e. with intact memory) will exhibit a preference for objects in a new location (object placement test) or a novel object (object recognition test) [18]. To measure depression-like behavior, the Porsolt swim test was used as previously described [18]. Briefly, mice were placed into a tank of water at room temperature. After an adjustment period of one minute, the amount of time spent immobile was measured as an indication of despair and depression-like behavior.

### RT-PCR

Snap-frozen skin was homogenized in Trizol using a Retsch MM300 Tissue Lyser to collect RNA. Chloroform was then added and the aqueous phase was collected and processed using the Agencourt RNAdvanced tissue kit that was modified for automation on a Biomek FXp from Beckman. RNA was quantified on a NanoDrop 8000 instrument and RNA quality was assessed based on RNA integrity numbers using the Agilent 2200 Tape Station. Reverse transcription was achieved using the TaqMan Reverse Transcription Reagents Kit (Applied Biosystems). The resultant cDNA was used in a ViiA 7 Real-Time PCR system (Applied Biosystems) using mouse-specific probes from Applied Biosystems.

### Cytokine protein expression

Protein was isolated from the skin using Tper buffer (Thermo Fisher Scientific, Waltham, MA, USA), and the isolated protein then used in Biolegend’s Legendplex Mouse Inflammation Panel (13-plex) per the manufacturer’s instructions.

### Flow cytometry

Brains were harvested from mice perfused with ice cold PBS, and then the choroid plexus of the third, fourth, and lateral ventricles was dissected out and combined. Choroid plexuses were infused with digestion buffer (2.5 mg/mL Liberase TL (Roche) and 1 mg/mL of DNase I (Roche) in Hank’s balanced salt solution (HBSS) plus magnesium and calcium), cut into small pieces and put into C-tubes (Miltenyi). C-tubes were positioned on a MACS dissociator and run on the m_brain_3 protocol, after which they were placed in an incubator for 30 minutes at 37 °C with shaking at 200 rpm. After incubation, C-tubes were positioned back on the MACS dissociator and run on the m_brain_3 protocol. The released cells were then passed through a 40-μm nylon filter with a cell masher, and filters were washed with 50 mL of wash buffer (1% BSA in HBSS plus magnesium and calcium). Cells were stained with a Fixable Viability Dye efluor 506 (eBioscience), incubated with Fc-Block (BD Bioscience) and stained with the appropriate fluorochrome-conjugated antibodies (Additional file 1: Table S1). Cell counts were determined using 123count eBeads Counting Beads according to the manufacturer’s instructions (eBioscience). Data were acquired on a BD LSR II flow cytometer (BD Biosciences, San Jose, CA, USA). Compensation and analysis of the flow cytometric data were performed using Flowjo software (TreeStar, Ashland, OR, USA). “Fluorescence minus one” controls were used when necessary to set up gates. The gating strategy is illustrated in Additional file 2: Figure S1.

### Quantitation of circulating IgG

Serum IgG levels were measured by ELISA, as previously described [8].

### Statistics and data analysis

Data were analyzed using Graphpad Prism with the appropriate statistical tests. Graphs were prepared using the same software. *P* values ≤ 0.05 were considered significant.

## Results

### BI-BTK-1 treatment prevents macroscopic skin pathology in MRL/lpr mice

**Fig. 1:**
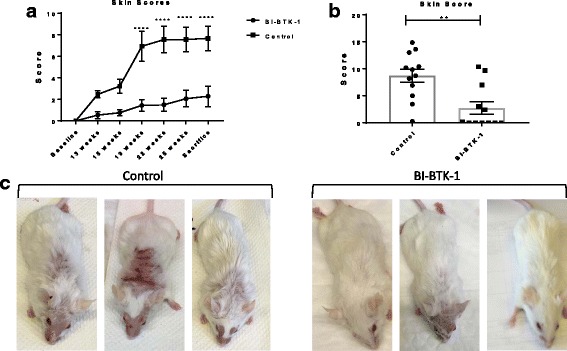
Cutaneous lesions in MRL/lpr mice. **a** Macroscopic lesions were scored over the course of the experiment up until the time of sacrifice (**b**). **c** BI-BTK-treated mice had ameliorated macroscopic skin lesions as compared to control-treated mice. Three representative mice are shown from each group. Shown are the results from one experiment (BI-BTK-1, *n* = 12; control, *n* = 12) (**p < 0.01, ****p < 0.0001)

MRL/lpr mice were treated with control chow or chow formulated with the BTK inhibitor, BI-BTK-1, starting at 8–9 weeks of age until the time of sacrifice (~25 weeks of age). BI-BTK-1 treatment significantly ameliorated the skin lesions seen in control mice by 19 weeks of age (Fig. 1a). Furthermore, this protection was maintained until the time of sacrifice, at which point only 5/12 (42%) of the BI-BTK-1 treated mice had any signs of skin disease, whereas 11/12 (92%) of the control mice had visible cutaneous involvement (*p* < 0.0001) (Fig. 1b). While some BI-BTK-1 treated mice still displayed alopecia or minor erythema, the skin appeared significantly healthier than in the control-treated counterparts (Fig. 1a, c). In contrast, control-treated mice developed severe macroscopic lesions characterized by alopecia, erythema, scaling, and thickening of the skin on both the face and dorsal thorax (Fig. 1c).

### BI-BTK-1 treatment significantly improves skin histopathology

**Fig. 2:**
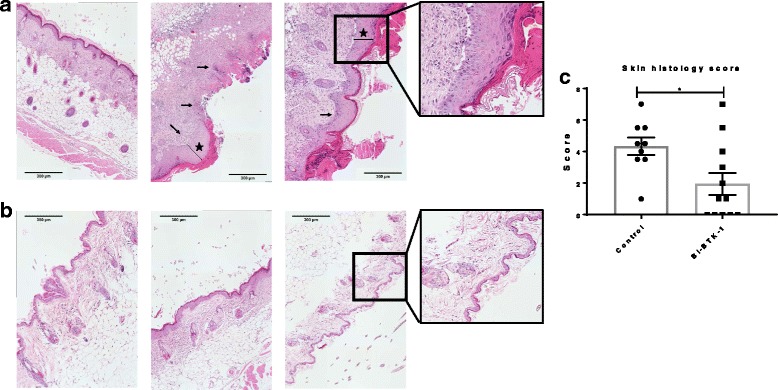
Skin histology. **a** Control-treated MRL/lpr mice at 26 weeks of age display severe inflammatory skin disease, as marked by cellular infiltration (small arrows) and hyperkerotosis (stars), which is markedly improved in BI-BTK-1-treated mice. **b** Representative images are taken at × 10 and show mice in the treated and control groups. The sections were blindly assessed and assigned a score. **c** BI-BTK-1, *n* = 12; control, *n* = 9 (*p < 0.05)

Control-treated MRL/lpr mice displayed histopathologic features of CLE, including thickening of the epidermis (hyperkeratosis) and cellular infiltration (Fig. 2a). In addition to alleviating macroscopic lesions, we found that treatment with BI-BTK-1 significantly improved cutaneous histopathology compared to control MRL/lpr mice (Fig. 2b). Evaluation of the blindly scored sections confirmed that BI-BTK-1 treated mice had significantly improved skin architecture compared to control mice (Fig. 2c).

### BI-BTK-1 treatment prevents immune cell accumulation in the skin

**Fig. 3:**
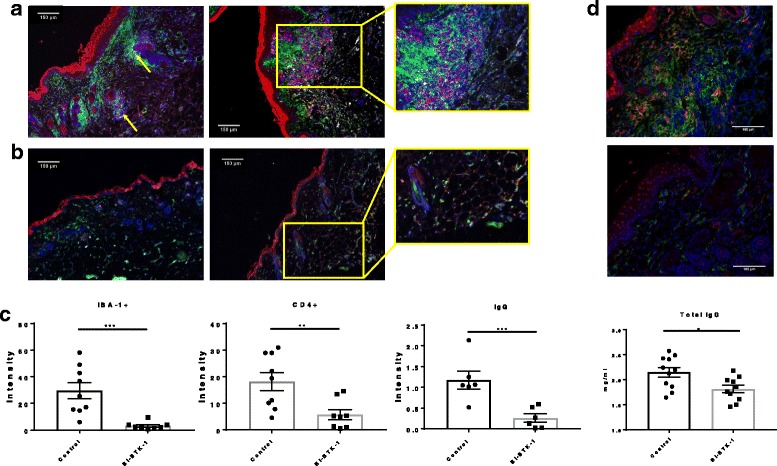
Cellular infiltration into the skin. **a** Control-treated MRL/lpr mice display prominent infiltration of the skin by macrophages (green) and T cells (red) (yellow arrows), and IgG deposition (gray), which is significantly reduced in BI-BTK-1-treated mice. **b** Representative images are taken at × 10 and show mice in the treated and control groups. **c** The intensities of the macrophage and T cell stains (BI-BTK-1, *n* = 8; control, *n* = 9), and the IgG stain (BI-BTK-1, *n* = 6; control, *n* = 6) were quantitated using ImageJ. Right panel, total circulating IgG concentrations were measured in terminal serum (BI-BTK-1, *n* = 10; control, *n* = 11; *p* < 0.05). **d** Sections from control (top panel) and BI-BTK-1-treated (bottom panel) mice were further co-stained with Mac-2 (red) and IBA-1 (green) to assess macrophage activation (× 20) (*p < 0.05, **p < 0.01, ***p < 0.001)

To further characterize the effects of BTK inhibition on spontaneous skin lesions in MRL/lpr mice, sections were stained for commonly infiltrating cells in CLE, namely macrophages (IBA-1^+^) and T cells (CD4^+^), to assess the effect of BTK inhibition on immune cell infiltration. Additionally, sections were stained for IgG to assess the effect of BI-BTK-1 on immunoglobulin deposition in the skin. While we found no significant difference in infiltration of B cells between the groups (data not shown), skin from BI-BTK-1 treated mice exhibited distinctly fewer macrophages, T cells, and IgG deposition compared to control mice (Fig. 3a and b). We also stained for neutrophils in the skin, but found no significant difference between the BI-BTK-1-treated and control-treated groups (data not shown). The intensity of each stain was measured using ImageJ, verifying that BI-BTK-1-treated mice had significantly diminished infiltration of both IBA-1+ macrophages and CD4+ T cells, and attenuated IgG deposition (Fig. 3c). Circulating serum IgG levels were also significantly decreased with BI-BTK-1 treatment (Fig. 3c, right panel). To further characterize the skin-infiltrating macrophages, sections were also stained with Mac-2, a macrophage activation marker. As can be seen in Fig. 3d, not only were there far larger numbers of IBA-1+ macrophages in the lesional skin of control mice, a large majority of them were Mac-2+ as well.

### BI-BTK-1 modulates expression of inflammatory mediators in the skin

**Fig. 4:**
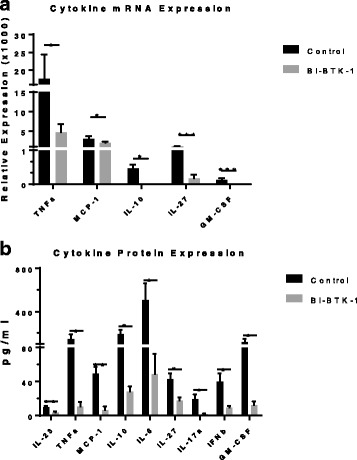
Inflammatory mediator expression in the skin. **a** RT-PCR was performed on mRNA isolated from lesional skin and assessed for the relative expression of various inflammatory cytokines. **b** Protein lysates were prepared from lesional skin, and cytokine expression quantitated as described in “Methods”. BI-BTK-1, *n* = 12; control, *n* = 9 (*p < 0.05, **p < 0.01, ***p < 0.001)

We assessed the relative expression of mRNA for various inflammatory mediators in the skin. As seen in Fig. 4a, control-treated MRL/lpr mice had increased TNF, monocyte chemoattract protein 1 (MCP-1), IL-10, IL-27, and granulocyte macrophage-colony stimulating factor (GM-CSF) as assessed by RT-PCR. However, treatment with BI-BTK-1 significantly reduced the expression of these inflammatory cytokines (Fig. 4a).

We also assessed the levels of various inflammatory cytokines in the skin at the protein level. As seen in Fig. 4b, BI-BTK-1 decreased the levels of a variety of cytokines previously associated with CLE pathogenesis [19], including TNF, IL-6, IL-17A, and IL-10. Additionally, the macrophage related cytokines MCP-1 and GM-CSF were also decreased by BI-BTK-1 treatment (Fig. 4b). Interferon-γ (IFNγ) and IL-1β levels, however, were not affected by BTK inhibition (data not shown).

### BI-BTK-1 treatment improves cognitive function in MRL/lpr mice

**Fig. 5:**
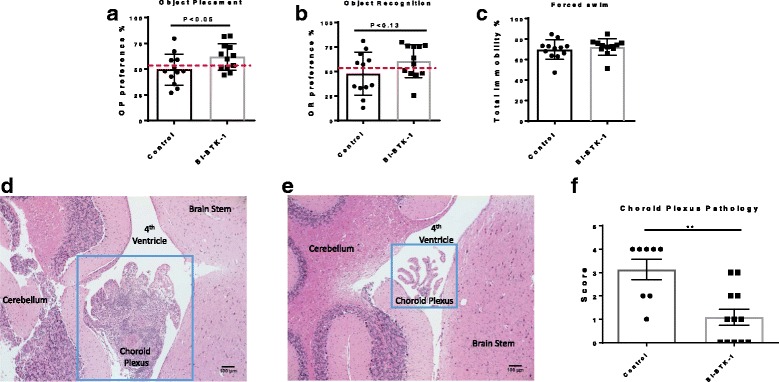
Neurobehavioral testing and choroid plexus infiltration. BI-BTK-1-treated mice have improved cognitive function as evidenced by significant improvement in the object placement test (**a**). A trend toward improvement in the object recognition test was present as well (**b**). Treatment had no effect on depression-like behavior (**c**) (BI-BTK-1, *n* = 12; control, *n* = 12). **d** MRL/lpr mice demonstrate marked choroid plexus infiltration with lymphocytes and macrophages, as seen here in a section of the fourth ventricle. Treatment with BI-BTK-1 ameliorates this infiltration (**e**). Representative images are taken at × 10 (**b** and **d**) and × 20 (**c**) and shown from mice in the treated and control groups, and blindly scored using the scale described in “Methods” (**f**). BI-BTK-1, *n* = 12; control, *n* = 8 (**p < 0.01)

MRL/lpr mice display cognitive dysfunction, including impaired visual and spatial memory, beginning around 16 weeks of age [18]. We performed behavioral testing on the BI-BTK-1 treated mice and control mice at 17 weeks of age, with mice subjected to both the object placement (spatial memory) and object recognition (visual memory) tests. Treated mice had a significant improvement in the object placement test, indicating preserved spatial memory (Fig. 5a); treated mice had a mean preference for exploration of the object at the novel location of 61 ± 13%, as compared to a preference of 50 ± 15% in the control mice (*p* < 0.05). A trend toward improvement was also observed in the object recognition test; however, this was not statistically significant (Fig. 5b).

MRL/lpr mice exhibit depression-like behavior as early as 5 weeks of age [18]. The effect of BI-BTK-1 treatment on this behavior was assessed at 17 weeks of age. We found that starting treatment with BI-BTK-1 at 8–9 weeks of age did not improve depression-like behavior, as assessed by the Porsolt forced swim test (Fig. 5c). Open field tests revealed that neither mouse group had gait deficits or musculoskeletal weakness, as both groups displayed similar total track lengths (data not shown). There were no differences in center track length, center time, or center visits, to indicate changes related to anxiety or risk-seeking behavior (data not shown).

### BI-BTK-1 treatment improves brain histologic appearance

We assessed H&E-stained brain sections to determine the effect of BI-BTK-1 treatment on infiltration of inflammatory cells into the central nervous system. Specifically, we focused on the choroid plexus, the site of the blood-cerebrospinal-fluid barrier, where we and others have previously described marked lymphocyte infiltration in the MRL/lpr strain starting at 16 weeks of age [20]. While control-treated MRL/lpr mice have prominent cellular infiltration into the choroid plexus in the region of dorsal fourth ventricle (Fig. 5d), BI-BTK-1-treated mice had marked diminution in the number of infiltrating cells (Fig. 5e). When the sections were blindly scored for stromal expansion and the degree of infiltrating immune cells, we found that BI-BTK-1 treatment significantly reduces choroid plexus pathology as compared to control mice (Fig. 5f). We also evaluated the presence of apoptosis within the cortex, choroid plexus, and hippocampus, but found no significant differences in the number of TUNEL-positive cells between BI-BTK-1-treated and control-treated mice (data not shown). Furthermore, there were no notable differences in markers of blood-brain barrier permeability, as assessed by staining for fibronectin, albumin, and IgG (data not shown).

### BI-BTK-1 reduces accumulation of T cells, B cells, and macrophages in the choroid plexus

**Fig. 6:**
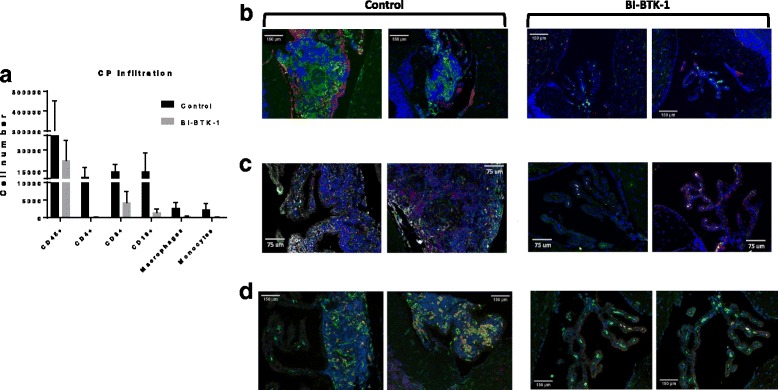
Characterization of the choroid plexus infiltrates. **a** Flow cytometric analysis of choroid plexus (CP) infiltrating immune cells in a small pilot study (BI-BTK-1, *n* = 2; control, *n* = 2) revealed infiltration of leukocytes, T cells, B cells, and macrophages. Immunofluorescent staining confirmed large populations of macrophages (green) accumulating in the choroid plexus of control mice (**b**), and increased IgG deposits (magenta) compared to BI-BTK-1-treated mice (**c**). Control mice also had increased numbers of T cells (red), B cells (green), and C3 complement deposition (gray). **d** Macrophages infiltrating the control-treated mice CPs had an activated phenotype (Mac-2+) compared to the BI-BTK-2-treated mice. Representative images are shown in all panels (BI-BTK-1, *n* = 7; control, *n* = 7)

To further investigate if the improvement in the neurobehavioral phenotype in BI-BTK-1-treated mice is associated with decreased brain infiltration by particular cell types, flow cytometry was performed on cells isolated from the choroid plexus of BI-BTK-1-treated and control-treated MRL/lpr mice age 17–18 weeks. We found that BI-BTK-1-treated mice exhibited significantly decreased infiltrating leukocytes (CD45+ cells), including CD4+ T cells, CD8+ T cells, and CD19+ B cells, as well as macrophages and monocytes (Fig. 6a).

To confirm the flow cytometric results in analyzing whether BI-BTK-1 was preventing the specific accumulation of particular types of immune cells in the choroid plexus, brain sections were stained for macrophages (IBA-1), T cells (CD4), and B cells (B220). We found that BTK inhibition prevented the accumulation of all three cell types (Fig. 6b, c). Furthermore, a decrease in locally deposited IgG and C3 was noted as well (Fig. 6b, c). We assessed the state of macrophage activation in the accumulating IBA-1+ cells by staining for Mac-2. As can be seen in Fig. 6d, not only do the control mice have more IBA-1+ macrophages but a larger percentage are Mac-2 positive, indicating a more inflammatory macrophage phenotype accumulating in the choroid plexus of control-treated mice. Finally, no meaningful accumulation of neutrophils was observed in the choroid plexus of either control-treated or BI-BTK-1-treated mice (data not shown).

## Discussion

Neuropsychiatric manifestations and skin involvement are two common and potentially serious end-organ pathologies associated with SLE, both of which present a pressing unmet therapeutic need. Current treatment still predominantly relies on broadly immunosuppressive medications, which while effective in some patients are associated with dangerous side effects and no guarantee of remission. In the study presented here, we showed that BI-BTK-1 treatment had dramatic effects in preventing the development of cutaneous lesions and ameliorating the cognitive dysfunction commonly seen in a classic lupus animal model. Whether or not BTK inhibition is better than existing treatments cannot be stated with confidence at this time, since our study design did not include an active comparator arm (e.g. treatment with high-dose steroids and/or cyclophosphamide). Moreover, although well beyond scope of the present study, future experiments will need to investigate the effect of delayed treatment on more established disease phenotypes, and on long-term survival. Nevertheless, narrowing the cells/pathways targeted in SLE is, at the very least, likely to improve the therapeutic index.

Considering the various cell types that have been implicated in the pathogenesis of SLE, therapeutic BTK inhibition would largely directly affect B cells and macrophages, both of which have been implicated in skin and brain disease. Although the precise mechanism underlying each individual type of end-organ pathology in lupus has yet to be fully elucidated, autoantibody deposition is thought to be important in both types of tissue [5, 21]. Other potential contributions of B cells to the mechanisms of target organ injury in lupus are antigen presentation and cytokine secretion [22]. Furthermore, we have previously shown that macrophages are important for the development of both spontaneous and ultraviolet B irradiation-induced skin lesions in lupus mice and in the pathogenesis of murine NPSLE [7–9].

Other investigators have previously reported the use of various BTK inhibitors in lupus models, including NZB/W, MRL/lpr, SLE1,3, and BXSB-Yaa strains and nephrotoxic serum nephritis (the latter a nephritis-limited model inducible in non-autoimmune mice following passive transfer of nephritogenic antibodies). In all cases, an improvement in kidney disease was reported [15, 23–26]. Hutchenson et al. also noted that in addition to the preserved kidney function in SLE1,3 mice treated with BTK inhibition there was also a decrease in autoreactive humoral immunity, with delayed production of autoantibodies and decreased splenomegaly. In NZB/W mice, BTK inhibition downregulated the expression of interferon related genes in the spleen, a finding attributed to decreased systemic activation of macrophages via their Fc receptors. These studies collectively show that BTK inhibition in lupus models can mitigate kidney disease. However, the effect of BTK inhibition on other central manifestations of murine (and human) lupus, including cutaneous and neuropsychiatric disease, has not been studied to date. These effects of BTK inhibition in antibody-mediated nephritis [15], and initial studies reporting good therapeutic results with reasonable safety in human disease (for non-lupus indications) [27], led us to the current study which was to focus the investigation on the effects of BTK inhibition in lupus-associated extra-renal involvement.

Skin lesions in MRL/lpr were significantly improved by BI-BTK-1 treatment, both macroscopically and histologically. Treated mice also had less cellular infiltration into the skin, and less IgG deposition; decreased numbers of both macrophages and T cells were seen, cell types which impact the lupus-associated cutaneous disease via effector functions and cytokine release. Further, the macrophages seen in BI-BTK-1-treated mice had a less activated phenotype. Indeed, BI-BTK-1 treatment decreased the concentration of skin cytokines, including several previously associated with CLE pathogenesis such as IL-6, IL-17, and TNF. BTK-dependent signaling is further known to promote macrophage polarization towards an inflammatory phenotype (M1) in vitro [13]. Consequently, decreased expression of these particular cytokines is likely to be mediated through preventing BTK-mediated macrophage polarization. Furthermore, BTK may also be important for downstream signaling of TLRs, specifically downstream of TLR4-dependent expression of IL-10 [14, 28]. The relevance of the latter mechanism here is supported by the decrease we found in skin IL-10 levels in BTK-I-treated mice. Finally, it is important to acknowledge that the less inflammatory cytokine environment present in treated mice may have indirectly contributed to attenuated macrophage activation. Nevertheless, our previous observation that BI-BTK-1 inhibits the secretion of IL-6, TNF, and IL-1 from isolated immune-complex-stimulated monocytes [15] is supportive of a conclusion that the reduced macrophage inhibition we observed here in vivo is at least partly due to the contribution of a direct inhibitory effect of BI-BTK-1 treatment.

We found that BI-BTK-1 treatment inhibited macrophage recruitment to inflamed skin. Studies in monocytes isolated from patients with mutated BTK have revealed that BTK defective monocytes have a decreased chemotactic response [29]. Further, we found here that BI-BTK-1 treatment also decreased MCP-1 and GM-CSF, both of which are important for macrophage recruitment and survival. Because macrophages can potentially secrete large amounts of cytokines, especially once activated by immunoglobulin deposited at the dermo-epidermal junction in CLE [1], decreased macrophage accumulation is likely a direct beneficial result of BI-BTK-1 treatment. BTK inhibition would also prevent macrophage activation via both Fc receptors (as we have previously shown [15]) and possibly through TLRs, both of which would be activated by autoantibodies or nuclear antigens deposited in the skin. Thus, the effect of BI-BTK-1 on macrophages could potentially be a major mechanism by which this drug ameliorates skin disease. The decrease in macrophage activation, and consequential decrease in inflammatory cytokine and chemokine expression, may contribute together to the decreased cellular infiltration seen in the skin, both for macrophages and T cells (even though the latter cell type is not directly impacted by BTK inhibition). Interestingly, Rankin [25] and Hutcheson [23] also found that BTK inhibition in lupus can affect the T cell compartment, and suggested that B cells are required for T cell maintenance [25]. In future studies, it would be interesting to isolate primary macrophages from BI-BTK-1-treated mice to carefully assess their phenotypes ex vivo and responses to various inflammatory stimuli.

We and others have previously demonstrated multiple effects by which BTK inhibition can modulate B cell function in lupus models. To briefly summarize some of the relevant effects, inhibition of BTK in vitro prevents B cell activation in response to BCR-mediated cross linking, and interferes with specific intracellular signaling pathways and anti-CD40-stimulated proliferation [24, 25, 30, 31]. In vivo, BTK inhibited spontaneous germinal center formation in lupus mice, and decreased splenic B cell numbers (marginal zone, follicular, B1, plasmablasts, and plasma cells), and circulating B cells, antibodies and/or autoantibodies [23–25, 31]. The magnitude of the effect observed and which B cell subset, antibody isotype, or specificity was most affected was at times variable between these studies, depending on the animal model used, drug administration protocol, and of course the actual molecule used for BTK inhibition. Nevertheless, it is clear that BTK treatment in lupus models is consistently associated with a marked effect on multiple B cell compartments and functions. Therefore, it is highly likely that the beneficial effects of BI-BTK-1 on skin and brain disease were brought about through its direct effects on B cell specific actions. Although it is possible that some B cell modulatory effects are more important than others, the significant benefit provided by BTK inhibition in lupus animal models is probably a function of multiple additive and/or synergistic effects on this key cell type.

In addition to the improvement in cutaneous disease, we found that BI-BTK-1 treatment improved NPSLE manifestations. Specifically, treated mice had improved cognitive function. Although the observed deficits in memory were not totally reversed, perhaps longer treatment would be required to demonstrate a more complete response. In this regard, the decreased accumulation of macrophages, T cells, and B cells in the choroid plexus was highly significant and very encouraging. BTK inhibition may inhibit lymphoid chemotaxis through the CCL20/CCR6 pathway, as both B cells and T cells are CCR6+, and expression of CCL20 is higher in the choroid plexus than elsewhere in the brain not only during experimental allergic encephalomyelitis but in normal mice as well [32].

BTK has also been shown to be important in neutrophil recruitment and function [33, 34]; in a model of sterile inflammation induced by focal hepatic necrosis, BTK deficiency decreased neutrophil recruitment to the location of injury [33]. In our study, however, we found no evidence of neutrophil infiltration into the choroid plexus, and in the skin we saw no differences in neutrophil numbers between the control and treated mice. These findings are consistent with the reported heterogeneity in the role of BTK in neutrophil physiology [35].

The macrophages in the brain, known as microglia, are a major source of inflammatory cytokines – which are also believed to be important in NPSLE pathogenesis [8]. Similar to what we discussed regarding the skin, BI-BTK-1 inhibition may have polarized the microglia (and/or brain infiltrating macrophages) to an M2-like phenotype, even in the presence of M1 driving stimuli. In the choroid plexus, decreased Mac-2 staining of IBA-1+ macrophages indicates less activation of these cells. Additionally, autoantibodies are also thought to contribute to NPSLE. BTK inhibition and its effect on B cells could potentially have affected this as well. Overall, BTK inhibition presents itself as a potentially valuable therapeutic option for NPSLE, in addition to any salutary effects on the skin disease.

## Conclusions

BTK inhibitors (e.g. ibrutinib) are already being used in human disease for hematologic indications (chronic lymphocytic leukemia and small lymphocytic lymphoma), highlighting the real translational potential of this pathway [27]. Ibrutinib is relatively well-tolerated in humans, with side effects including upper respiratory tract infection, fatigue, and diarrhea, although none so severe to require its discontinuation [27]. BI-BTK-1 was designed to have improved selectivity and potency over existing inhibitors [15], potentially minimizing the side effects currently seen. Overall, we believe BI-BTK-1 may hold significant promise for the treatment of resistant SLE manifestations, including brain and skin disease.

## Additional file

Additional file 1: Table S1. Antibodies utilized for choroid plexus flow cytometric analysis. (DOCX 12 kb)
Additional file 2: Figure S1. Flow cytometry gating strategy. Cortical and choroid plexus tissue from MRL/lpr mice treated or not with BI-BTK-1 were analyzed by flow cytometric analysis. Red arrows denote sequential gated population (red boxes). Black arrows denote sequential non-gated population. (DOCX 358 kb)

## Abbreviations

BCR: B cell receptor
BSA: Bovine serum albumin
BTK: Burton’s tyrosine kinase
CLE: Cutaneous lupus erythematosus
ELISA: Enzyme-linked immunosorbent assay
H&E: Hematoxylin and eosin
IL: Interleukin
MCP-1: Monocyte chemoattract protein 1
NPSLE: Neuropsychiatric lupus
PBS: Phosphate-buffered saline
SLE: Systemic lupus erythematosus
TLR: Toll-like receptor
TNF: Tumor necrosis factor
