# Supplementary material for: Highly selective inhibition of Bruton’s tyrosine kinase attenuates skin and brain disease in murine lupus
Source: Arthritis Res Ther. 2018 Jan 25;20:10. doi: 10.1186/s13075-017-1500-0 (PMC5785891; doi:10.1186/s13075-017-1500-0)
Supplement: Additional file 1: Table S1. — Antibodies utilized for choroid plexus flow cytometric analysis. (DOCX 12 kb) [file 13075_2017_1500_MOESM1_ESM.docx]

**Table S1.** Antibodies utilized for choroid plexus flow cytometric analysis

| **Antigen** | **Clone** | **Fluorochrome** | **Manufacturer** |
| --- | --- | --- | --- |
| CD45 | 30-F11 | FITC | eBioscience |
| MHC II | M5/114.15.2 | PerCP-Cy5.5 | Biolegend |
| CD8 | 53-6.7 | efluor 450 | eBioscience |
| CD64 | X54-5/7.1 | APC | Biolegend |
| CD11b | M1/70 | APC-Cy7 | BD Biosciences |
| CD19 | 1D3 | PE | BD Biosciences |
| Siglec F | E50-2440 | PE-CF594 | BD Biosciences |
| Ly6G | 1A8 | PE-CF594 | BD Biosciences |
| NK1.1 | PK136 | PE-CF594 | BD Biosciences |
| CD4 | RM4-5 | Alexa700 | BD Biosciences |
| CD11c | HL3 | PE-Cy7 | BD Biosciences |
