# Supplementary material for: Highly selective inhibition of Bruton’s tyrosine kinase attenuates skin and brain disease in murine lupus
Source: Arthritis Res Ther. 2018 Jan 25;20:10. doi: 10.1186/s13075-017-1500-0 (PMC5785891; doi:10.1186/s13075-017-1500-0)
Supplement: Additional file 2: Figure S1. — Flow cytometry gating strategy. Cortical and choroid plexus tissue from MRL/lpr mice treated or not with BI-BTK-1 were analyzed by flow cytometric analysis. Red arrows denote sequential gated population (red boxes). Black arrows denote sequential non-gated population. (DOCX 358 kb) [file 13075_2017_1500_MOESM2_ESM.docx]

A. Cortex

B. Chroid Plexus

**Figure S1**. Flow cytometry gating strategy. Cortical and choroid plexus tissue from MRL/lpr mice treated or not with BI-BTK-1 were analyzed by flow cytometric analysis. Red arrows denote sequential gated population (red boxes). Black arrows denote sequential non-gated population.
